# Reliability of routinely collected anthropometric measurements in primary care

**DOI:** 10.1186/s12874-019-0726-8

**Published:** 2019-04-24

**Authors:** Sarah Carsley, Patricia C. Parkin, Karen Tu, Eleanor Pullenayegum, Nav Persaud, Jonathon L. Maguire, Catherine S. Birken, on behalf of the TARGet Kids! Collaboration, Catherine S. Birken, Jonathon L. Maguire, Eddy Lau, Andreas Laupacis, Patricia C. Parkin, Michael Salter, Peter Szatmari, Shannon Weir, Laura N. Anderson, Cornelia M. Borkhoff, David W. H. Dai, Christine Kowal, Dalah Mason, Murtala Abdurrahman, Barbara Anderson, Kelly Anderson, Gordon Arbess, Jillian Baker, Tony Barozzino, Sylvie Bergeron, Dimple Bhagat, Nicholas Blanchette, Gary Bloch, Joey Bonifacio, Ashna Bowry, Anne Brown, Jennifer Bugera, Caroline Calpin, Douglas Campbell, Sohail Cheema, Elaine Cheng, Brian Chisamore, Evelyn Constantin, Erin Culbert, Karoon Danayan, Paul Das, Mary Beth Derocher, Anh Do, Michael Dorey, Kathleen Doukas, Anne Egger, Allison Farber, Amy Freedman, Sloane Freeman, Sharon Gazeley, Charlie Guiang, Dan Ha, Shuja Hafiz, Curtis Handford, Laura Hanson, Leah Harrington, Hailey Hatch, Teresa Hughes, Sheila Jacobson, Lukasz Jagiello, Gwen Jansz, Mona Jasuja, Paul Kadar, Tara Kiran, Lauren Kitney, Holly Knowles, Bruce Kwok, Sheila Lakhoo, Margarita Lam-Antoniades, Eddy Lau, Fok-Han Leung, Alan Li, Patricia Li, Jennifer Loo, Joanne Louis, Sarah Mahmoud, Jessica Malach, Roy Male, Vashti Mascoll, Aleks Meret, Rosemary Moodie, Julia Morinis, Maya Nader, Katherine Nash, Sharon Naymark, James Owen, Jane Parry, Michael Peer, Kifi Pena, Marty Perlmutar, Navindra Persaud, Andrew Pinto, Michelle Porepa, Vikky Qi, Nasreen Ramji, Noor Ramji, Jesleen Rana, Danyaal Raza, Alana Rosenthal, Katherine Rouleau, Janet Saunderson, Rahul Saxena, Vanna Schiralli, Michael Sgro, Hafiz Shuja, Susan Shepherd, Barbara Smiltnieks, Cinntha Srikanthan, Carolyn Taylor, Suzanne Turner, Fatima Uddin, Meta van den Heuvel, Joanne Vaughan, Thea Weisdorf, Sheila Wijayasinghe, Peter Wong, Anne Wormsbecker, Ethel Ying, Elizabeth Young, Michael Zajdman, Farnaz Bazeghi, Vincent Bouchard, Marivic Bustos, Charmaine Camacho, Dharma Dalwadi, Christine Koroshegyi, Tarandeep Malhi, Sharon Thadani, Julia Thompson, Laurie Thompson, Mary Aglipay, Imaan Bayoumi, Sarah Carsley, Katherine Cost, Karen Eny, Theresa Kim, Laura Kinlin, Jessica Omand, Shelley Vanderhout, Leigh Vanderloo, Christopher Allen, Bryan Boodhoo, Olivia Chan, Judith Hall, Peter Juni, Gerald Lebovic, Karen Pope, Kevin Thorpe, Rita Kandel

**Affiliations:** 1 Institute of Health Policy, Management and Evaluation, Dalla Lana School of Public Health, University of Toronto, Toronto, Canada; 2 Division of Paediatric Medicine, Department of Paediatrics, Child Health Evaluative Sciences, Peter Gilgan Centre for Research and Learning, the Hospital for Sick Children, Room 109801, 10th Floor, 686 Bay Street, Toronto, ON M5G 0A4 Canada; 3 Division of Pediatric Medicine and the Pediatric Outcomes Research Team (PORT), Department of Pediatrics, University of Toronto Faculty of Medicine, Toronto, Canada; 4 Department of Family and Community Medicine, University of Toronto, Toronto, Canada; 5 Toronto Western Hospital Family Health Team, University Health Network, Toronto, Canada; 6 Department of Family Medicine, St. Michael’s Hospital and Li Ka Shing Knowledge Institute, Toronto, Canada; 7 Department of Pediatrics, St. Michael’s Hospital and Li Ka Shing Knowledge Institute, Toronto, Canada; 8 Department of Nutritional Sciences, University of Toronto, Toronto, Canada

**Keywords:** Growth, Measurement, Height, Weight, Reliability, TEM, Childhood obesity

## Abstract

**Background:**

Measuring body mass index (BMI) has been proposed as a method of screening for preventive primary care and population surveillance of childhood obesity. However, the accuracy of routinely collected measurements has been questioned. The purpose of this study was to assess the reliability of height, length and weight measurements collected during well-child visits in primary care relative to trained research personnel.

**Methods:**

A cross-sectional study of measurement reliability was conducted in community pediatric and family medicine primary care practices. Each participating child, ages 0 to 18 years, was measured four consecutive times; twice by a primary care team member (e.g. nurses, practice personnel) and twice by a trained research assistant. Inter- and intra-observer reliability was calculated using the technical error of measurement (TEM), relative TEM (%TEM), and a coefficient of reliability (R).

**Results:**

Six trained research assistants and 16 primary care team members performed measurements in three practices. All %TEM values for intra-observer reliability of length, height, and weight were classified as ‘acceptable’ (< 2%; range 0.19% to 0.70%). Inter-observer reliability was also classified as ‘acceptable’ (< 2%; range 0.36% to 1.03%) for all measurements. Coefficients of reliability (R) were all > 99% for both intra- and inter-observer reliability. Length measurements in children < 2 years had the highest measurement error. There were some significant differences in length intra-observer reliability between observers.

**Conclusion:**

There was agreement between routine measurements and research measurements although there were some differences in length measurement reliability between practice staff and research assistants. These results provide justification for using routinely collected data from selected primary care practices for secondary purposes such as BMI population surveillance and research.

**Electronic supplementary material:**

The online version of this article (10.1186/s12874-019-0726-8) contains supplementary material, which is available to authorized users.

## Background

Growth measurement during the first years of life is essential for optimizing child health [1, 2]. Primary care practitioners use these measurements to guide parents and caregivers when children fall outside healthy growth parameters. Weight, height and length measurements are used as growth indicators to calculate body mass index-for-age which has been recommended as the most inexpensive, efficient and precise measure in primary care practice to determine if a child has overweight or obesity [3]. However, imprecise measurements may lead to misclassification of weight status, unnecessary interventions, referrals and patient and parental concern [4]. The World Health Organization (WHO), and Dietitians of Canada recommend that measurement techniques be standardized; length for children less than 2 years of age measured in the recumbent position and standing height for children older than 2 years age. Further, equipment should be calibrated with those responsible for measurement trained for accuracy and reliability [1]. These procedures for growth monitoring have been previously used in research studies such as the Canadian Health Measures Survey [5] in Canada and the National Health and Nutrition Examination Survey (NHANES) in the United States [6].

All growth measurements are subject to error which can be random or systematic and may occur from human error or equipment error [7]. Reliability is the extent “to which within-subject variability is due to factors other than measurement error variance or physiological variation” [8–10]. The lower the variability between repeated measurements of the same subject the greater the precision [10]. There are two forms of reliability that will be evaluated in this study: intra-observer and inter-observer reliability. Intra-observer reliability is the ability for one observer (the term observer will be used to define the person measuring the subject) to repeat measurements on the same child with little to no variability. Inter-observer reliability is the ability for two independent observers to measure the same child with little or no variability. Determining both intra-and inter-observer reliability is important in evaluating the accuracy of measurement data. The accuracy of routinely collected anthropometric data is currently unknown relative to measurements taken by trained personnel. Imprecise measurements can weaken observed associations of exposure and health outcomes both clinically and for research. The purpose of this study was to assess the reliability of height, length and weight measurements collected during well-child visits in primary care practices. A secondary objective was to determine any systematic differences in intra-observer reliability between primary care team members and research assistants.

## Methods

### Study setting and population

Parents or guardians of healthy children 0 to 18 years attending a scheduled well-child visit were invited to participate and informed consent was obtained. Children were recruited at two pediatric practices and one family medicine primary care practice participating in the TARGet Kids! [11] research network in Toronto, Canada (www.targetkids.ca). Based on TARGet Kids! exclusion criteria at enrollment, children were ineligible to participate if they were diagnosed with associated health conditions affecting growth (such as failure to thrive or cystic fibrosis) or if their parent or guardian was not fluent in English. Primary care team members, including nurses and clinic staff at the 3 practices volunteered to perform the routine care measurements. This study was approved by the Research Ethics Boards of the Hospital for Sick Children and St. Michael’s Hospital.

### Sampling and sample size calculation

A convenience sample was used to recruit participants until the required sample size was achieved in each age group: 0 to < 2 years, 2- < 5 years, and 5–18 years. Children younger than 2 years of age and 2 to 5 years were over sampled because length and height measurements have been shown to be particularly variable in these age groups [12, 13]. The sample sizes per age category was determined based on previous work by Walter and colleagues using 4 replicates (measurements per subject) [14]. In the age groups 0 to 2 years, with α = 0.05 and β = 0.2 (corresponding to 80% power) to rule out the possibility of a reliability < 0.7 (the minimally acceptable value), and to achieve an expected reliability coefficient (R) of at least 0.8 the number of subjects required was 68. In the age groups 2 to 5 years, to achieve the expected reliability coefficient (R) of 0.85 the number of subjects required was 26. In the age group 5 to 18 years to achieve the expected reliability coefficient (R) of at least 0.9, the number of subjects required was 12.

### Data collection and measurement

Research measurements were performed by a research assistant; routine measurements were performed by a primary care team member, both on the same equipment. Research assistants were trained using the WHO Training Course Growth Assessment modules [15]. These growth monitoring guidelines were adopted by multiple professional health agencies such as the Canadian Pediatric Society, and Community Health Nurses of Canada and are the guidelines primary care team members would have received in their professional training [16]. Primary care team members did not receive any further training other than what they received during their professional degrees or at the practice where they worked. The standard procedure and equipment for measurement of length in children < 2 years was in the recumbent position using a length board (SECA model#2101821009 length board), and weight of infants was measured without clothing or diapers on a digital baby scale (Healthometer 553 KL pediatric scale). Measurement of height and weight in children ≥2 years was performed in light clothes without shoes on a digital scale with standing height attachment (Healthometer 500 KL adult scale). In total, each participating child had their age and sex recorded, and 4 sets of anthropometric measurements (4 weights and 4 lengths/heights), with each observer performing the measurements twice. The first observer measured weight and length/height, and recorded the measurements on a standardized data collection form. The child was then measured by the second observer and measurements recorded on a separate form. The process was repeated to obtain a second reading from both the first and second observer. In order to limit each observer’s recall of their previous measurements, the observers alternated and recorded measurements on separate forms. The order of the observer who performed the first measurement was random, so that the research assistant and the primary care team member could be either first or second observer. Each observer was blind to the other observer’s measurements.

### Statistical analysis

Descriptive statistics were used to describe the children included in this study. Intra-observer reliability was assessed by comparing each observer’s first measurement to their own second measurement. Inter-observer reliability was assessed by comparing the initial observer’s first measurement to the second observer’s first measurement and by comparing second measurements therefore using all four measurements from each independent observer. Measures of central tendency (mean, median, and mode) were calculated for the absolute difference between measurements by age group. Bland-Altman plots were used to describe the differences between and within observers graphically. Infants 0 to < 2 years were calculated separately because they were measured using different equipment. Intra- and inter-observer reliability statistics were calculated for children 0 to 2 years, 2 to 5 years and > 5 to 18 years. Intra-observer reliability statistics for each observer and Bland Altman plots were calculated for children 2 to 18 years to maximize sample size. The technical error of measurement (TEM), the relative TEM (%TEM), and the coefficient of reliability (R) were the statistical tests used to assess intra- and inter-observer reliability. The TEM was defined as the standard deviation of differences between repeated measures in the unit of the measurement (e.g. TEM for height measured in centimeters is cm), using the following equation:

$$ TEM=\sqrt{\sum {D}^2/2N} $$

Where *D* is the difference between repeated measures and *N* is the number of individuals measured. TEMs with lower values indicate greater precision of the observer performing the measurement [10]. The relative TEM was calculated as the (TEM/mean × 100).

*R*, the coefficient of reliability is the estimated proportion of inter-subject variance that is not due to measurement error, defined by the equation:

$$ R=1-\left({TEM}^2/{SD}^2\right) $$

SD^2^ is the total inter-subject variance for the study population. Scores vary from 0 to 1, with 0 indicating that all between subject variations are due to measurement error and a value of 1 indicating that no measurement error is present. Higher R values are indicative of greater precision, with values above 0.95 considered acceptable, 0.8 considered sufficient and values lower than 0.7 considered minimally acceptable measurement error [10]. All three reliability measurements (TEM, %TEM, and R) were calculated to compare with published reliability statistics. Finally, an F-statistic was calculated to test differences between intra-observer reliabilities by squaring the technical error of measurement to create a variance and dividing one by another: F = TEM^2^_(intra1)_/TEM^2^_(intra2)_ with degrees of freedom = N-1. A significance level of 0.05 was used to determine statistical significance [17].

## Results

### Sample characteristics

In total 125 children were recruited and measured 4 times, contributing 498 weight measurements and 500 length or height measurements. These measurements were performed by 6 trained research assistants (RA) from the TARGet Kids! research network and 16 primary care team members. Additional file 1: Table S1 shows the proportion of measurements each observer contributed to the study. Summary statistics of the subject characteristics are presented in Table 1. The median age was 19 months (IQR 9.0 to 53.0 months); there were 68 children (54.4%) < 2 years, 31 (24.8%) between 2 and 5 years, and 26 (20.8%) > 5 to 18 years. Boys and girls were almost equally represented, 50.4 and 49.6%, respectively. The majority of infants and children had a normal weight (between − 2 and ≤ 1 BMI z-score), 6.5% had a ‘risk of overweight’ (zBMI between 1 and ≤ 2) and 5.7% had ‘overweight’ status (zBMI ≥2). One subject < 2 years became agitated and only completed 1 set of weight measurements; therefore the sample to calculate inter- and intra-observer reliability in this age group was decreased by 2 and 1, respectively.

**Table 1:** Characteristics of sample population

Measurement by age group	N	Mean	Std Dev	Minimum	Q1	Median	Q3	Maximum
Age (months)	125	33.6	32.2	0.23	9.0	19.0	53.0	135.0
Sex, Male (%)	63 (50.4)							
0- < 2 years								
Weight (kg)	67^a^	8.66	1.92	2.51	7.29	8.65	9.69	13.37
Length (cm)	68	71.6	7.57	45.63	66.53	71.46	76.85	86.38
2 to 5 years								
Weight (kg)	31	15.36	2.61	11.58	13.1	14.53	17.7	19.78
Height (cm)	31	99.58	8.51	83.9	93.18	100.53	107.8	110.78
> 5 to 18 years								
Weight (kg)	26	24.02	5.73	15.65	20.4	22.6	27.23	37.55
Height (cm)	26	124.34	11.03	102.85	116.35	123.93	130.05	146.4

^a^1 patient did not complete all weight measurements

### Inter-observer reliability

**Fig. 1:**
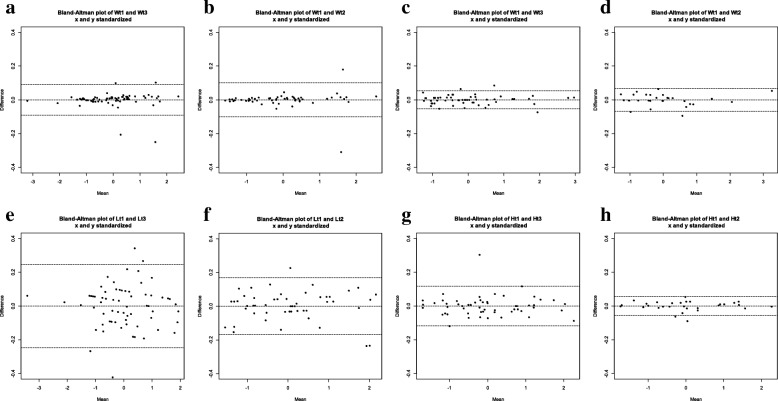
Bland-Altman plots on the weight and length/height measurements for intra- and inter-observer reliability by age group. Top row of plots: **a**) inter-observer reliability for weight 0- < 2 years, **b**) intra-observer reliability weight 0- < 2, **c**) inter-observer reliability for weight 2–18 years, **d**) intra-observer reliability for weight 2–18 years, **e**) inter-observer reliability for length 0- < 2 years, **f**) intra-observer reliability for length 0- < 2 years, **g**) inter-observer reliability height 2–18 years, **h**) intra-observer reliability for height 2–18 years

Intra- and inter-observer reliability for each measurement type by age group is presented in Table 2. The absolute mean difference for weight ranged from 0.03–0.15 kg and 0.52–0.77 cm for length/height. Overall, all %TEM values for weight, length and height were in the acceptable range of < 2% [18] and coefficients of reliability (R) values were all > 99%, representing very good reliability between repeated measurements performed by two independent observers [10]. Inter-observer reliability of length, < 2 years, had the highest TEM (0.73 cm) and a %TEM of 1.03%. Relative TEM for weight slightly increased as child age increased from 0.64% in children < 2 years to 0.70% in children > 5 years. In contrast, the %TEM for length/height improved as child age increased from 1.03% to 0.36%. Figure 1 shows the inter- and intra-observer differences in weight, length/height by age group (< 2 and ≥ 2 years) using Bland-Altman plots. The majority of differences were within 2 standard deviations of the mean, considered acceptable levels of error. The largest differences were length measurements of children < 2 years, and the smallest differences were weight measurements of children < 2 years.

**Table 2:** Intra and inter-observer reliability statistics by measurement type and age group

Measurement	N	Absolute Mean Difference	TEM	%TEM	R
Inter-observer reliability					
Weight (kg)					
0- < 2 years	270	0.03	0.06	0.64	0.9992
2–5 years	124	0.10	0.10	0.65	0.9985
> 5 to 18 years	104	0.15	0.17	0.70	0.9991
Length/Height (cm)					
0- < 2 years	272	0.77	0.73	1.03	0.9906
2–5 years	124	0.58	0.69	0.70	0.9933
> 5 to 18 years	104	0.52	0.45	0.36	0.9983
Intra-observer reliability					
Weight (kg)					
0- < 2 years	135	0.03	0.05	0.61	0.9992
2–5 years	62	0.09	0.10	0.62	0.9987
> 5 to 18 years	52	0.16	0.17	0.70	0.9991
Length/Height (cm)					
0- < 2 years	136	0.46	0.46	0.64	0.9963
2–5 years	62	0.39	0.27	0.27	0.9990
> 5 to 18 years	52	0.35	0.24	0.19	0.9995

### Intra-observer reliability

The absolute mean difference for weight ranged from 0.03–0.16 kg and 0.35–0.46 cm for length/height. In general, intra-observer reliability was more precise compared to inter-observer reliability. Relative TEM for weight ranged from 0.61% to 0.70%, and 0.19% to 0.64% for length/height. R values were all > 99%, representing very good reliability of each observer between their own repeated measurements. In further analyses, intra-observer reliabilities were calculated separately for the RA observers and the primary care practitioners with the highest proportion of measurements and tested for statistically significant differences (Tables 3 and 4). TEMs for weight ranged from 0.02–0.20 cm, for length TEMs 0.35-0.63 cm, and for height TEMs were 0.38–0.47 cm. Statistical differences between RA and primary care practitioners were seen in the weight and length intra-observer reliabilities in children < 2 years in one of the pediatric practices; the RA had lower TEMs for length and the primary care practitioner had a slightly higher TEM for weight. In the family medicine practice, length measurements taken by the RA had a lower TEM than the primary care nurse. Further data on measurement differences and calculations are presented in Additional file 2: Table S2 and Additional file 3: Table S3.

**Table 3:** Intra-observer results for weight and length/height measurements by individual observers

Observer	0 to < 2 years			2 to 18 years		
	WEIGHT			WEIGHT		
	TEM (kg)	%TEM	R	TEM (kg)	%TEM	R
Pediatric Clinics						
Research assistant 1	0.06	0.72%	0.9987	0.15	0.51%	0.9997
Primary care nurse 1	0.04	0.46%	0.9993	0.20	1.03%	0.9991
Research assistant 2				0.09	0.48%	0.9998
Primary care nurse 2				0.12	0.56%	0.9998
Family Medicine Clinic						
Research assistant 3	0.02	0.20%	0.9998	0.18	0.90%	0.9977
Primary care nurse 3	0.02	0.26%	0.9997	0.10	0.51%	0.9991
	LENGTH			HEIGHT		
	TEM (cm)	%TEM	R	TEM (cm)	%TEM	R
Pediatric Clinics						
Research assistant1	0.43	0.60%	0.9958	0.53	0.34%	0.9993
Primary care nurse 1	0.63	0.91%	0.9863	0.47	0.42%	0.9992
Research assistant 2				0.34	0.30%	0.9997
Primary care nurse 2				0.46	0.41%	0.9993
Family Medicine Clinic						
Research assistant3	0.06	0.35%	0.9976	0.21	0.19%	0.9996
Primary care nurse 3	0.45	0.62%	0.9915	0.38	0.33%	0.9987

**Table 4:** Differences in intra-observer reliability technical error of measurement (TEM) between TARGet Kids! research assistants and primary care nurses

Practice	Measurement	Research		Routine Care			
		TEM	df	TEM	df	F-Statistic	*p*-value
Pediatric Clinic 1	Wt 0–2	0.06	54	0.04	21	2.25	0.022*
	Lt 0–2	0.43	54	0.63	21	2.14	0.013*
	Wt 2–18	0.15	29	0.20	16	0.56	0.913
	Ht 2–18	0.53	29	0.47	16	1.27	0.313
Pediatric Clinic 2	Wt 2–18	0.09	18	0.12	12	0.56	0.869
	Ht 2–18	0.34	18	0.46	12	0.55	0.881
Family Medicine Clinic	Wt 0–2	0.02	7	0.02	3	1.00	0.553
	Lt 0–2	0.06	7	0.45	3	56.25	< 0.001*
	Wt 2–18	0.18	5	0.10	4	3.24	0.139
	Ht 2–18	0.21	5	0.38	4	0.31	0.887

*Significant *p*-value < 0.05

## Discussion

This study reports the intra- and inter-observer reliability of weight, length and height measurements performed in one family medicine and two pediatric practices using multiple reliability statistics such as the technical error of measurement (TEM) and the coefficient of reliability (R). All %TEM values were < 2% and R coefficients > 99% meaning both intra- and inter-observer reliability were acceptable and had high reliability. This supports the acceptability of using routinely collected weight and length/height measurements from these primary care practices.

Our findings are similar to previous studies that performed measurement reliability testing for quality assurance purposes involving multiple anthropometrists in large epidemiologic studies. For example, the World Health Organization Multicentre Growth Reference Standards (WHO-MGRS) [19], NHANES [17], Born in Bradford [8], and the Identification and prevention of Dietary- and lifestyle-induced health Effects In Children and Infants (IDEFICS) [9] study have published reliability data on the observers involved in measurement. In each of these studies all TEMs and R values for length/height and weight were in an acceptable range and indicative of good quality. In the WHO-MGRS expert anthropometrists were used as a gold standard and had intra-observer TEM of 0.29 cm for length and 0.23 cm for height (weight was not included). Although our TEM for length was higher (0.46 cm), our TEMs for height were comparable at 0.27 cm (2- < 5 years) and 0.24 cm (5+ years). In one review of reliability statistics mean intra-observer TEMs for length, height and weight were 0.35 cm, 0.38 cm, and 0.17 kg, respectively, which were similar to our study [10]. One main difference of these studies is test-retest measurements were all performed after the anthropometrists had undergone standardized training. In our study, we did not provide the primary care practitioners any further training other than what they received in their clinical training as our objective was to assess reliability in routine primary care.

Length had the highest %TEM for inter-observer reliability which is consistent with previous studies that have shown increased measurement error in length [13, 20]. Observing the Bland-Altman plots, differences in length measurements show greater spread. One possible explanation relates to the challenge in positioning of very young infants appropriately on the length board. In our study, the %TEMs for height were better for older children who may be easier to position on the stadiometer. Conversely, the results for weight were better for younger children, although the difference in %TEM between each age group was minimal (< 0.1%). This may be due to the consistent use of digital scales in all the practices. Moreover, very young infants tend to move less on the baby scale as opposed to preschool-aged children (2 to 5 years) who may have difficulty standing still or may not be in the exact center of the scale.

There were limitations to this study. Not as many children were recruited from the family medicine practice compared to the pediatric practices because of the higher volume of children seen for well-baby care in the latter. As well, the practices that volunteered to participate were already participating in research through TARGet Kids!, therefore may have had more standardized protocols related to measurement compared to other primary care practices in Ontario. We were unable to measure reliability at one of the pediatric clinics for children under 2 years. Both primary care team members and research assistants were aware of the purpose of this study therefore may have changed their measurement behaviour. Finally, there were multiple primary care practitioners contributing to the overall intra-observer reliability TEMs for each measure which may have inflated the estimates. However, since this was intended as a pragmatic examination of primary care anthropometry we included all data collected by all observers.

While this study was able to assess reliability of human measurements, we were not able to assess the potential measurement error resulting from equipment or measurement methods. For example assessing the use of a length board rather than the paper and pencil method where a marking is made on the examining table paper at the head and feet. This method has been shown to systematically overestimate length thereby increasing measurement error [21].

## Conclusion

Monitoring weight, height and length measurements to calculate BMI-for-age has been recommended as the most inexpensive, efficient and precise measure in primary care to assess weight status. With the increased use of electronic medical records (EMR), this data is accessible for use outside of clinical care such as public health surveillance. In this study, we assessed multiple observers to calculate both intra- and inter-observer reliability and demonstrated all values were in the acceptable range. Although length measurement had the highest TEM, it was still acceptable according to standards based on both published reliability statistics [18] and comparable to the expert anthropometrists from the WHO-MGRS [19], meaning the magnitude of human measurement error was small. Determining the measurement reliability of length/height and weight in primary care contributes to understanding the feasibility of using routine clinical data for BMI surveillance in children. This study has identified that primary care practitioners in selected primary care practices who adhere to standardized equipment and procedures measure weight, length/height as well as research trained personnel.

## Additional files

Additional file 1 **Table S1.** Percentage of measurements performed by each observer by age group. This table presents the number and percent of how many study participants were measured by each observer: research assistants (RA) and primary care team member (PCTM). (DOCX 19 kb)

Additional file 2 **Table S2.** TEM and R calculations of intra- and inter-observer reliability for length and height. This table presents the differences in length/height measurements observed by the research assistants and primary care team members by age of participant and the calculations for summary statistics (mean, median, mode), the technical error of measurement, and coefficient of reliability. (DOCX 25 kb)

Additional file 3 **Table S3.** TEM and R calculations of intra- and inter-observer reliability for weight. This table presents the differences in weight measurements observed by the research assistants and primary care team members by age of participant and the calculations for summary statistics (mean, median, mode), the technical error of measurement, and coefficient of reliability. (DOCX 24 kb)

## Abbreviations

BMI: Body Mass Index
R: Coefficient of Reliability
TEM: Technical Error of Measurement
