# Supplementary material for: Reliability of routinely collected anthropometric measurements in primary care
Source: BMC Med Res Methodol. 2019 Apr 24;19:84. doi: 10.1186/s12874-019-0726-8 (PMC6480730; doi:10.1186/s12874-019-0726-8)
Supplement: Supplementary file 1 — Table S1. Percentage of measurements performed by each observer by age group. This table presents the number and percent of how many study participants were measured by each observer: research assistants (RA) and primary care team member (PCTM). (DOCX 19 kb) [file 12874_2019_726_MOESM1_ESM.docx]

**Supplemental Table 1:** Percentage of measurements performed by each observer

| **Clinic** | **Research Assistant (RA)** | **Observer** | **Total** | | **0-<2 years** | | **2-5 years** | | **>5-18 years** | |
| --- | --- | --- | --- | --- | --- | --- | --- | --- | --- | --- |
|  | **Primary Care Team Member (PCTM)** |  | **N** | **%** | **N** | **%** | **N** | **%** | **N** | **%** |
| Pediatric 1 | RA | 1 | 85 | 68.0 | 55 | 80.9 | 18 | 58.1 | 12 | 46.2 |
|  | PCTM | 2 | 39 | 31.2 | 22 | 32.4 | 8 | 25.8 | 9 | 34.6 |
|  | PCTM | 3 | 24 | 19.2 | 17 | 25.0 | 5 | 16.1 | 2 | 7.7 |
|  | PCTM | 4 | 2 | 1.6 | 2 | 2.9 | 1 | 3.2 | 1 | 3.8 |
|  | PCTM | 5 | 17 | 13.6 | 12 | 17.6 | 4 | 12.9 | 1 | 3.8 |
|  | PCTM | 6 | 1 | 0.8 | 1 | 1.5 | 0 | 0.0 | 0 | 0.0 |
|  | PCTM | 7 | 2 | 1.6 | 1 | 1.5 | 1 | 3.2 | 0 | 0.0 |
| Pediatric 2 | RA | 8 | 19 | 15.2 | 0 | 0.0 | 9 | 29.0 | 10 | 38.5 |
|  | PCTM | 9 | 13 | 10.4 | 0 | 0.0 | 5 | 16.1 | 8 | 30.8 |
|  | PCTM | 10 | 3 | 2.4 | 0 | 0.0 | 2 | 6.5 | 1 | 3.8 |
|  | PCTM | 11 | 2 | 1.6 | 0 | 0.0 | 2 | 6.5 | 0 | 0.0 |
|  | PCTM | 12 | 1 | 0.8 | 0 | 0.0 | 0 | 0.0 | 1 | 3.8 |
| FHT | RA | 13 | 14 | 11.2 | 8 | 11.8 | 3 | 9.7 | 3 | 11.5 |
|  | RA | 14 | 4 | 3.2 | 3 | 4.4 | 0 | 0.0 | 0 | 0.0 |
|  | RA | 15 | 1 | 0.8 | 1 | 1.5 | 0 | 0.0 | 0 | 0.0 |
|  | RA | 16 | 2 | 1.6 | 1 | 1.5 | 1 | 3.2 | 0 | 0.0 |
|  | PCTM | 17 | 8 | 6.4 | 3 | 4.4 | 2 | 6.5 | 3 | 11.5 |
|  | PCTM | 18 | 6 | 4.8 | 4 | 5.9 | 0 | 0.0 | 2 | 7.7 |
|  | PCTM | 19 | 3 | 2.4 | 3 | 4.4 | 0 | 0.0 | 0 | 0.0 |
|  | PCTM | 20 | 1 | 0.8 | 1 | 1.5 | 0 | 0.0 | 0 | 0.0 |
|  | PCTM | 21 | 1 | 0.8 | 0 | 0.0 | 1 | 3.2 | 0 | 0.0 |
|  | PCTM | 22 | 2 | 1.6 | 1 | 1.5 | 0 | 0.0 | 1 | 3.8 |
