# Supplementary material for: Reliability of routinely collected anthropometric measurements in primary care
Source: BMC Med Res Methodol. 2019 Apr 24;19:84. doi: 10.1186/s12874-019-0726-8 (PMC6480730; doi:10.1186/s12874-019-0726-8)
Supplement: Supplementary file 2 — Table S2. TEM and R calculations of intra- and inter-observer reliability for length and height. This table presents the differences in length/height measurements observed by the research assistants and primary care team members by age of participant and the calculations for summary statistics (mean, median, mode), the technical error of measurement, and coefficient of reliability. (DOCX 25 kb) [file 12874_2019_726_MOESM2_ESM.docx]

**Supplemental Table 2:** TEM and R calculations of intra- and inter-observer reliability for length and height

| **Length (cm) 0-2** | | | | | | **Height (cm) 2 to 5 years** | | | | | | **Height (cm) >5 to 18** | | | | | |
| --- | --- | --- | --- | --- | --- | --- | --- | --- | --- | --- | --- | --- | --- | --- | --- | --- | --- |
|  |  | Inter-observer | | Intra-observer | |  |  | Inter-observer | | Intra-observer | |  |  | Inter-observer | | Intra-observer | |
| Di | Di^2 | N | % | N | % | Di | Di^2 | N | % | N | % | Di | Di^2 | N | % | N | % |
| 0.0 | 0.00 | 55 | 20.22 | 46 | 33.82 | 0.0 | 0.00 | 14 | 11.29 | 18 | 29.03 | 0.0 | 0.00 | 15 | 14.42 | 15 | 28.85 |
| 0.1 | 0.01 | 2 | 0.74 | 1 | 0.74 | 0.1 | 0.01 | 9 | 7.26 | 6 | 9.68 | 0.1 | 0.01 | 11 | 10.58 | 4 | 7.69 |
| 0.2 | 0.04 | 10 | 3.68 | 5 | 3.68 | 0.2 | 0.04 | 17 | 13.71 | 5 | 8.06 | 0.2 | 0.04 | 11 | 10.58 | 5 | 9.62 |
| 0.3 | 0.09 | 17 | 6.25 | 13 | 9.56 | 0.3 | 0.09 | 16 | 12.9 | 2 | 3.23 | 0.3 | 0.09 | 12 | 11.54 | 7 | 13.46 |
| 0.5 | 0.25 | 63 | 23.16 | 31 | 22.79 | 0.4 | 0.16 | 10 | 8.06 | 4 | 6.45 | 0.4 | 0.16 | 7 | 6.73 | 5 | 9.62 |
| 0.6 | 0.36 | 2 | 0.74 | 1 | 0.74 | 0.5 | 0.25 | 23 | 18.55 | 9 | 14.52 | 0.5 | 0.25 | 13 | 12.5 | 5 | 9.62 |
| 0.7 | 0.49 | 9 | 3.31 | 7 | 5.15 | 0.6 | 0.36 | 4 | 3.23 | 4 | 6.45 | 0.6 | 0.36 | 4 | 3.85 | 3 | 5.77 |
| 0.8 | 0.64 | 8 | 2.94 | 1 | 0.74 | 0.7 | 0.49 | 7 | 5.65 | 4 | 6.45 | 0.7 | 0.49 | 4 | 3.85 | 1 | 1.92 |
| 0.9 | 0.81 | 1 | 0.37 | 0 | 0 | 0.8 | 0.64 | 4 | 3.23 | 1 | 1.61 | 0.8 | 0.64 | 6 | 5.77 | 1 | 1.92 |
| 1.0 | 1.00 | 43 | 15.81 | 20 | 14.71 | 0.9 | 0.81 | 4 | 3.23 | 2 | 3.23 | 0.9 | 0.81 | 1 | 0.96 | 0 | 0.00 |
| 1.1 | 1.21 | 0 | 0.00 | 1 | 0.74 | 1.0 | 1.00 | 5 | 4.03 | 3 | 4.84 | 1.0 | 1.00 | 4 | 3.85 | 5 | 9.62 |
| 1.2 | 1.44 | 5 | 1.84 | 0 | 0 | 1.1 | 1.21 | 0 | 0.00 | 1 | 1.61 | 1.1 | 1.21 | 2 | 1.92 | 0 | 0.00 |
| 1.3 | 1.69 | 3 | 1.10 | 0 | 0 | 1.2 | 1.44 | 1 | 0.81 | 1 | 1.61 | 1.2 | 1.44 | 3 | 2.88 | 0 | 0.00 |
| 1.5 | 2.25 | 20 | 7.35 | 9 | 6.62 | 1.3 | 1.69 | 0 | 0.00 | 2 | 3.23 | 1.3 | 1.69 | 2 | 1.92 | 0 | 0.00 |
| 1.6 | 2.56 | 1 | 0.37 | 0 | 0 | 1.4 | 1.96 | 2 | 1.61 | 0 | 0 | 1.4 | 1.96 | 1 | 0.96 | 0 | 0.00 |
| 1.7 | 2.89 | 3 | 1.10 | 1 | 0.74 | 1.5 | 2.25 | 1 | 0.81 | 0 | 0 | 1.5 | 2.25 | 2 | 1.92 | 0 | 0.00 |
| 1.8 | 3.24 | 4 | 1.47 | 0 | 0 | 1.6 | 2.56 | 1 | 0.81 | 0 | 0 | 1.6 | 2.56 | 4 | 3.85 | 0 | 0.00 |
| 2.0 | 4.00 | 15 | 5.51 | 0 | 0 | 1.8 | 3.24 | 2 | 1.61 | 0 | 0 | 1.7 | 2.89 | 1 | 0.96 | 0 | 0.00 |
| 2.5 | 6.25 | 6 | 2.21 | 0 | 0 | 3.9 | 15.21 | 2 | 1.61 | 0 | 0 | 1.8 | 3.24 | 1 | 0.96 | 0 | 0.00 |
| 2.8 | 7.84 | 2 | 0.74 | 0 | 0 | 4.9 | 24.01 | 2 | 1.61 | 0 | 0 | 2.1 | 4.41 | 0 | 0.00 | 1 | 1.92 |
| 3.0 | 9.00 | 3 | 1.10 | 0 | 0 |  |  |  |  |  |  |  |  |  |  |  |  |
| Sample size, n | | 272 | 100 | 136 | 100 |  |  | 124 | 100 | 62 | 100 |  |  | 104 | 100 | 52 |  |
| Mean Di (cm) | | 0.77 |  | 0.46 |  |  |  | 0.58 |  | 0.39 |  |  |  | 0.52 |  | 0.35 |  |
| Median (cm) | | 0.5 |  | 0.5 |  |  |  | 0.4 |  | 0.35 |  |  |  | 0.4 |  | 0.3 |  |
| Mode (cm) | | 0.5 |  | 0.0 |  |  |  | 0.5 |  | 0.0 |  |  |  | 0.0 |  | 0.0 |  |
| Sum Di^2 | | 293.4 |  | 57.9 |  |  |  | 120.3 |  | 18.0 |  |  |  | 51 |  | 14.5 |  |
| Sum Di^2/2n | | 0.539 |  | 0.21 |  |  |  | 0.485 |  | 0.07 |  |  |  | 0.21 |  | 0.058 |  |
| TEM (cm) | | 0.734 |  | 0.46 |  |  |  | 0.694 |  | 0.27 |  |  |  | 0.45 |  | 0.242 |  |
| %TEM | | 1.026 |  | 0.64 |  |  |  | 0.70 |  | 0.27 |  |  |  | 0.36 |  | 0.194 |  |
| R | | 0.9906 | | 0.9963 | |  |  | 0.9933 | | 0.9990 | |  |  | 0.9983 | | 0.9995 | |
