# Supplementary material for: Reliability of routinely collected anthropometric measurements in primary care
Source: BMC Med Res Methodol. 2019 Apr 24;19:84. doi: 10.1186/s12874-019-0726-8 (PMC6480730; doi:10.1186/s12874-019-0726-8)
Supplement: Supplementary file 3 — Table S3. TEM and R calculations of intra- and inter-observer reliability for weight. This table presents the differences in weight measurements observed by the research assistants and primary care team members by age of participant and the calculations for summary statistics (mean, median, mode), the technical error of measurement, and coefficient of reliability. (DOCX 24 kb) [file 12874_2019_726_MOESM3_ESM.docx]

**Supplemental Table 3:** TEM and R calculations of intra- and inter-observer reliability for weight

| **Weight (kg) 0-2 years** | | | | | | **Weight (kg) 2 to 5 years** | | | | | | **Weight (kg) >5 to 18 years** | | | | | |
| --- | --- | --- | --- | --- | --- | --- | --- | --- | --- | --- | --- | --- | --- | --- | --- | --- | --- |
|  |  | **Inter-observer** | | **Intra-observer** | |  |  | **Inter-observer** | | **Intra-observer** | |  |  | **Inter-observer** | | **Intra-observer** | |
| Di | Di^2 | N* | % | N** | % | Di | Di^2 | N | % | N | % | Di | Di^2 | N | % | N | % |
| 0.00 | 0.00 | 75 | 27.78 | 34 | 25.19 | 0.0 | 0.00 | 48 | 38.71 | 27 | 43.55 | 0.0 | 0.00 | 40 | 38.46 | 13 | 25.00 |
| 0.01 | 0.00 | 67 | 24.81 | 52 | 38.52 | 0.1 | 0.01 | 46 | 37.1 | 22 | 35.48 | 0.1 | 0.01 | 29 | 27.88 | 22 | 42.31 |
| 0.02 | 0.00 | 44 | 16.3 | 18 | 13.33 | 0.2 | 0.04 | 20 | 16.13 | 8 | 12.9 | 0.2 | 0.04 | 15 | 14.42 | 7 | 13.46 |
| 0.03 | 0.00 | 31 | 11.48 | 10 | 7.41 | 0.3 | 0.09 | 8 | 6.45 | 3 | 4.84 | 0.3 | 0.09 | 2 | 1.92 | 2 | 3.85 |
| 0.04 | 0.00 | 8 | 2.96 | 3 | 2.22 | 0.4 | 0.16 | 0 | 0.00 | 2 | 3.23 | 0.4 | 0.16 | 9 | 8.65 | 4 | 7.69 |
| 0.05 | 0.00 | 14 | 5.19 | 6 | 4.44 | 0.5 | 0.25 | 2 | 1.61 | 0 | 0.00 | 0.5 | 0.25 | 6 | 5.77 | 1 | 1.92 |
| 0.06 | 0.00 | 6 | 2.22 | 2 | 1.48 |  |  |  |  |  |  | 0.6 | 0.36 | 1 | 0.96 | 2 | 3.85 |
| 0.07 | 0.00 | 2 | 0.74 | 1 | 0.74 |  |  |  |  |  |  | 0.8 | 0.64 | 1 | 0.96 | 1 | 1.92 |
| 0.08 | 0.01 | 2 | 0.74 | 2 | 1.48 |  |  |  |  |  |  | 0.9 | 0.81 | 1 | 0.96 | 0 | 0.00 |
| 0.09 | 0.01 | 2 | 0.74 | 0 | 0.00 |  |  |  |  |  |  |  |  |  |  |  |  |
| 0.10 | 0.01 | 4 | 1.48 | 1 | 0.74 |  |  |  |  |  |  |  |  |  |  |  |  |
| 0.11 | 0.01 | 3 | 1.11 | 1 | 0.74 |  |  |  |  |  |  |  |  |  |  |  |  |
| 0.14 | 0.02 | 1 | 0.37 | 0 | 0.00 |  |  |  |  |  |  |  |  |  |  |  |  |
| 0.16 | 0.03 | 2 | 0.74 | 0 | 0.00 |  |  |  |  |  |  |  |  |  |  |  |  |
| 0.17 | 0.03 | 1 | 0.37 | 0 | 0 |  |  |  |  |  |  |  |  |  |  |  |  |
| 0.18 | 0.03 | 0 | 0.00 | 1 | 0.74 |  |  |  |  |  |  |  |  |  |  |  |  |
| 0.23 | 0.05 | 1 | 0.37 | 0 | 0.00 |  |  |  |  |  |  |  |  |  |  |  |  |
| 0.24 | 0.06 | 1 | 0.37 | 1 | 0.74 |  |  |  |  |  |  |  |  |  |  |  |  |
| 0.29 | 0.08 | 0 | 0.00 | 1 | 0.74 |  |  |  |  |  |  |  |  |  |  |  |  |
| 0.39 | 0.15 | 2 | 0.74 | 0 | 0 |  |  |  |  |  |  |  |  |  |  |  |  |
| 0.42 | 0.18 | 2 | 0.74 | 1 | 0.74 |  |  |  |  |  |  |  |  |  |  |  |  |
| 0.52 | 0.27 | 2 | 0.74 | 0 | 0 |  |  |  |  |  |  |  |  |  |  |  |  |
| 0.57 | 0.32 | 0 | 0.00 | 1 | 0.74 |  |  |  |  |  |  |  |  |  |  |  |  |
| Sample size, n | | 270 | 100 | 135 | 100 |  |  | 124 | 100 | 62 | 100 |  |  | 104 | 100 | 52 | 100 |
| Mean Di (kg) | | 0.03 |  | 0.03 |  |  |  | 0.097 |  | 0.09 |  |  |  | 0.15 |  | 0.16 |  |
| Median (kg) | | 0.01 |  | 0.01 |  |  |  | 0.1 |  | 0.1 |  |  |  | 0.1 |  | 0.1 |  |
| Mode (kg) | | 0.00 |  | 0.01 |  |  |  | 0.0 |  | 0.0 |  |  |  | 0.0 |  | 0.1 |  |
| Sum Di^2 | | 1.6447 |  | 0.7636 |  |  |  | 2.48 |  | 1.13 |  |  |  | 5.82 |  | 2.93 |  |
| Sum Di^2/2N | | 0.0031 |  | 0.003 |  |  |  | 0.01 |  | 0.01 |  |  |  | 0.028 |  | 0.03 |  |
| TEM | | 0.055 |  | 0.0531 |  |  |  | 0.1 |  | 0.095 |  |  |  | 0.167 |  | 0.168 |  |
| %TEM | | 0.637 |  | 0.614 |  |  |  | 0.651 |  | 0.621 |  |  |  | 0.696 |  | 0.699 |  |
| R | | 0.9992 |  | 0.9992 |  |  |  | 0.9985 |  | 0.9987 |  |  |  | 0.9991 |  | 0.9991 |  |

*Frequency Missing = 2 **Frequency Missing = 1
